# Electrically Conductive Natural Rubber Composite Films Reinforced with Graphite Platelets

**DOI:** 10.3390/polym16020288

**Published:** 2024-01-20

**Authors:** Veerapat Kitsawat, Saranrat Siri, Muenduen Phisalaphong

**Affiliations:** Bio-Circular-Green Economy Technology & Engineering Center, BCGeTEC, Department of Chemical Engineering, Faculty of Engineering, Chulalongkorn University, Bangkok 10330, Thailand; kit.veerapat@gmail.com (V.K.); saranrat13030@gmail.com (S.S.)

**Keywords:** alginate, composites, electrical conductivity, graphite, latex, natural rubber

## Abstract

Green natural rubber (NR) composites reinforced with synthetic graphite platelets, using alginate as a thickening and dispersing agent, were successfully developed to improve mechanical properties, chemical resistance, and electrical conductivity. The fabrication was performed using a latex aqueous microdispersion process. The research demonstrated the effective incorporation of graphite platelets into the NR matrix up to 60 parts per hundred rubbers (phr) without causing agglomeration or phase separation. Graphite incorporation significantly improved the mechanical strength of the composite films. NR with 60 phr of graphite exhibited the highest Young’s modulus of 12.3 MPa, roughly 100 times that of the neat NR film. The reinforcement also strongly improved the hydrophilicity of the composite films, resulting in a higher initial water absorption rate compared to the neat NR film. Moreover, the incorporation of graphite significantly improved the chemical resistance of the composite films against nonpolar solvents, such as toluene. The composite films exhibited biodegradability at about 21% to 30% after 90 days in soil. The electrical conductivity of the composite films was considerably enhanced up to 2.18 × 10^−4^ S/cm at a graphite loading of 60 phr. According to the improved properties, the developed composites have potential applications in electronic substrates.

## 1. Introduction

The excessive accumulation of plastic waste, including the widespread distribution of microplastics in the oceans, has become a major global concern. This has generated interest in the development of biodegradable materials to replace non-biodegradable petroleum-based products [1]. Natural rubber (NR) is an incredibly versatile polymer that is widely used in various industries. It is a biopolymer of *cis*-1,4-polyisoprene, containing numerous double bonds in its structure [2,3]. Thailand has been known as the world’s largest NR producer and exporter [4]. The production of NR primarily originates from *Hevea Brasiliensis*, commonly known as the Para rubber tree. It is extensively utilized in the production of various rubber products, including automotive tires, gloves, condoms, clothes, threads, adhesives, medical devices, and more. When NR is exported, it usually undergoes processing to create intermediate products, such as ribbed smoke sheets (RSS), un-smoked sheets (USS), block rubber (standard Thai rubber, STR), concentrated latex, compound rubber, and crepe rubber. However, Thailand sometimes is confronted by NR problems, such as fluctuating and unstable pricing caused by supply and demand imbalances [5].

Although NR exhibits many exceptional properties, including high flexibility, elasticity, excellent tear strength, toughness, and abrasion resistance, it also has limitations in terms of poor chemical resistance, limited temperature usage due to temperature sensitivity caused by environmental factors such as weather, sunlight, and ozone exposure [6,7,8]. The incorporation of fillers has been shown to be an effective strategy to improve the properties of NR. Fillers are substances that serve to enhance specific attributes of the material. Among them, carbonaceous fillers have received significant amounts of attention for their ability to enhance the electrical conductivity, thermal conductivity, fire resistance, and mechanical properties of polymers [9,10,11,12]. Common carbonaceous fillers include graphite (G), graphene (GP), carbon black (CB), fullerenes, carbon fibers (CF), and carbon nanotubes (CNT) [13].

Graphite is a microscale crystalline carbon allotrope commonly employed in the fabrication of polymer composites. It can be obtained naturally or synthetically. The structure of graphite is made up of parallel layers of graphene sheets with carbon atoms organized in a hexagonal pattern via strong covalent bonds. Van der Waals forces hold the parallel sheets together, giving the material anisotropic properties [14,15,16]. The π orbitals and free electrons are indeed evenly distributed throughout the graphene layers, which contributes to its exceptional electrical and thermal conductivity. Furthermore, graphite offers the advantages of being chemically and thermally stable, cost-effective, readily available, and versatile [17]. These factors make it appealing for various applications on commercial scales.

Both graphite and NR are hydrophobic or non-polar materials. However, due to graphite’s high density, surface energy, and limited wettability, it is challenging to disperse it uniformly in the NR matrix [18,19,20]. In most cases, rubber composites are produced using the melt compounding technique, employing an internal mixer and a two-roll mill. However, when mixing small-sized fillers, there is a possibility of producing an inconsistent mixture or encountering agglomerated fillers. To address these challenges, this research employs the process of latex aqueous microdispersion to achieve more effective distribution and dispersion of graphite in the NR matrix.

Alginate is a biopolymer derived primarily from brown seaweed. It has captured researchers’ attention due to its easy availability, non-toxic nature, biocompatibility, gel-forming property, and ability to degrade naturally [21,22]. When incorporated into NR, it offers a range of benefits, such as improving biodegradability, water-retention properties, processability, and both chemical and thermal stability [22,23,24]. Moreover, it acts as an efficient dispersing and thickening agent, increasing the viscosity of the rubber matrix. This elevated viscosity minimizes agglomeration and sedimentation, leading to improved filler dispersion and enhancing the uniformity and stability of the composites. Previous studies demonstrated that alginate could facilitate the mixing of various fillers in NR matrices [25,26,27,28,29]. This insight encourages our exploration into incorporating alginate to disperse graphite platelets, aiming to produce uniform composite films with high graphite loading. Alginate is anticipated to prevent the restacking and agglomeration of graphite platelets.

The objective of this study is to fabricate unvulcanized NR composite films incorporated with synthetic graphite platelets, which was facilitated by the addition of alginate as a thickening and dispersing agent. The effects of graphite as a reinforcing agent on the mechanical and thermal properties, hydrophilicity, chemical resistance, electrical conductivity, and biodegradability of NR composite films were investigated. The analysis involves comparing the properties of the composite films with different graphite loading as well as evaluating their performance against neat NR films.

## 2. Materials and Methods

### 2.1. Material

Natural rubber latex (NRL) with 60% dry rubber content (DRC) was purchased from the Rubber Research Institute of Thailand, Bangkok, Thailand. Synthetic graphite in the platelet form with a density of 2.26 g/cm^3^ and a particle size of less than 20 µm was purchased from Sigma-Aldrich (Buchs, Switzerland). The sodium salt of alginic acid (sodium alginate, ALG) was purchased from Acros Organics (Thermo Fisher Scientific Co., Ltd., Geel, Belgium).

### 2.2. Fabrication of Natural Rubber, Graphite, and Alginate (NR-G-ALG) Composite Films

**Figure 1:**
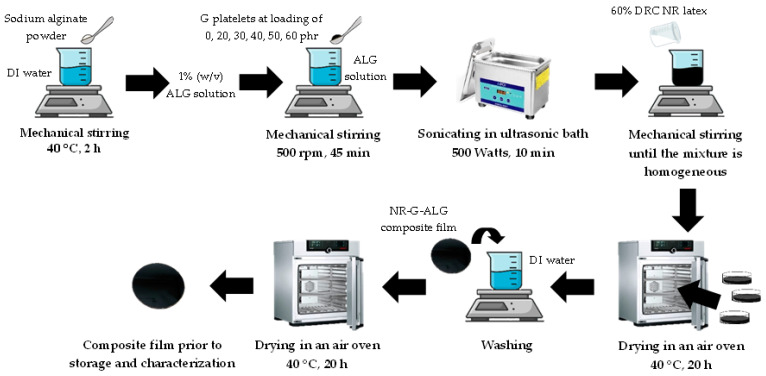
The illustrative schematic diagram for the fabrication of NR-G-ALG composite films.

The amount of graphite used in this study varied at 0, 20, 30, 40, 50, and 60 phr, corresponding to weight percentages of the composite film (wt.% of 0, 15.4, 21.4, 26.7, 31.3, and 35.3, respectively. The preparation of composite films was carried out using the latex aqueous microdispersion process, as previously reported by our research group [25,26,30,31,32]. The alginate solution was prepared by dispersing ALG powder in deionized water. The mixture was mechanically stirred and heated at 40 °C for 2 h until complete dissolution of ALG powder, resulting in a 1% *w/v* ALG solution. Afterward, graphite was added into 30 mL ALG solution and thoroughly mixed the slurry under mechanical stirring at 500 rpm for 45 min. Subsequently, the slurry was exposed to sonication in ultrasonic cleaner at 500 watts for 10 min to further disperse the filler particles and ensure homogeneity of the mixture. Following this, 5 g of 60% DRC NRL was gradually added into the slurry while continuously mixing by mechanical stirring until the homogenous mixture was obtained. Thereafter, the mixture was poured into a glass Petri dish and dried at 40 °C for 20 h in an air-circulating oven to obtain NR composite films. Afterward, the composite films were washed with deionized water to remove any impurities, followed by drying in an oven at 40 °C to eliminate residual moisture. The composite films were stored in an airtight container prior to characterization. The composite films of NR combined with graphite and ALG were denoted as NR-Gx-ALG, where x represents the content of graphite. For instance, the label NR-G20-ALG indicates the incorporation of 20 phr of graphite in the composite film. The illustrative schematic diagram for the fabrication of NR-G-ALG composite films is shown in Figure 1.

### 2.3. Characterization

The functional groups and possible polymer–filler interaction in the NR and NR composite films were analyzed using a Fourier transform infrared spectrometer (FTIR) (Spectrum One, Perkin Elmer, Shelton, CT, USA) by examining wavenumbers from 4000 to 400 cm^−1^ with a resolution of 4 cm^−1^. FTIR spectra were acquired in Transmission mode.

The morphology of G platelets, the surface and cross-section of the composite films, and the distribution of platelets in the composite films were examined using a field-emission scanning electron microscope (FE-SEM) (Quanta 250 FEG, Hillsboro, OR, USA) equipped with an energy-dispersive X-ray spectroscopy system. The dried sample films were coated with a thin layer of gold using a JEC-1100E sputter coater and imaged at an accelerating voltage of 15 kV. The average size of graphite platelets was determined using ImageJ 1.54d software.

The crystallinity and structural information of G platelets and the composite films were investigated using an X-ray diffractometer (XRD) (Bruker D8 Discover, Karlsruhe, Germany) with Cu-Kα radiation in the 2θ range of 5–60°. The operation conditions included an accelerating voltage of 40 kV and an electric current of 40 mA. The degree of crystallinity was calculated using the formula below.

(1) $$\mathrm{Degree}\;\mathrm{of}\;\mathrm{Crystallinity}\;(\%)=\frac{\mathrm{Crystalline}\;\mathrm{area}\times 100}{\mathrm{Total}\;\mathrm{area}}$$

The mechanical properties of the NR and NR composite films, including tensile strength, Young’s modulus, and elongation at break, were evaluated using a universal testing machine (UTM H10 KM, Redhill, UK) in accordance with the ASTM D882 standard [33]. The rectangular specimens with dimensions of 6 × 0.5 cm^2^ were placed between the upper and lower jaws of the instrument, and a controlled tensile force was gradually applied until the point of breakage. At least five specimens were tested for each composition to ensure statistical significance.

The thermal stability of the NR, G, and NR composite films, which refers to their ability to resist thermal degradation assessed by measuring weight loss, was tested via thermogravimetric analysis (TGA) (NETZSCH STA 449 F3 Jupiter, Selb, Germany). The test was conducted in a nitrogen atmosphere with a temperature range from 30 to 700 °C and a heating rate of 10 °C/min. The sample weights employed in the test ranged from 20 to 30 mg.

The thermal properties of the NR, G, and NR composite films, such as the melting temperature (T_m_), degradation temperature (T_d_), and glass transition temperature (T_g_) of the polymer, were analyzed using differential scanning calorimetry (DSC) (NETZSCH DSC 204 F1 Phoenix, Selb, Germany). The samples were heated in a nitrogen atmosphere from −100 to 300 °C at a rate of 10 °C/min, and thermal transitions, whether endothermic or exothermic, were recorded. The sample weights employed in the test ranged from 5 to 15 mg.

Water absorption capacity (WAC) serves as an indicator of the composite films’ ability to interact with water molecules, which corresponds to the water-retention capabilities of the composite films. To measure WAC, the sample films were cut into 2 × 2 cm^2^ and immersed in deionized water at room temperature. After a certain period of time, the films were removed from the water, and excess water at the surface was wiped off using KIMWIPES™ Delicate Task Wipers (Kimberly-Clark Corporation, Irving, TX, USA). The weight of the wet films was then measured, and the procedure was repeated until no further weight change was observed. At least three specimens were tested for each composition. WAC was calculated by using the formula where W_w_ and W_d_ represent the weight of wet and dry specimen films, respectively, as follows:

(2) $$\mathrm{WAC}\;(\%)=\frac{W_{w}-W_{d}}{W_{d}}\times 100\%$$

Toluene uptake (TU) serves as an indicator of the composite films’ capacity to absorb the non-polar solvent, toluene. This characteristic relates to the films’ chemical resistance. To measure TU, the sample films were cut into 2 × 2 cm^2^ and immersed in toluene at room temperature. After a certain period of time, the films were removed from toluene. The weight of the swollen films was measured, and the procedure was repeated until no further weight change was observed. At least three specimens were tested for each composition. The TU value was calculated using the formula where W_b_ and W_a_ represent the weight of the specimen films before and after immersion in toluene, respectively, as follows:

(3) $$\mathrm{TU}\;(\%)=\frac{W_{a}-W_{b}}{W_{b}}\times 100\%$$

The biodegradability test of the NR and NR composite films in soil was conducted for a duration of 3 months. Each composition of the composite films was cut into 5 × 5 cm^2^ and buried in soil flowerpots at a depth of 10 cm below the surface under ambient weather and climate conditions. The soil used was loam soil, primarily composed of coconut coir and composted raintree leaves. The moisture content in the soil was maintained within the range of 30 to 60% during the experiments. The samples were meticulously excavated and weighed after 1, 2, and 3 months of burial. For weight loss calculation, the excavated samples were rinsed and washed with deionized water and then dried in an air-circulating oven at 40 °C for 24 h before being weighed. The biodegradability rate of each composition was evaluated by the following equation, where W_f_ is the weight of the films after being excavated from the soil, and W_i_ is the weight of the films before being buried in the soil.

(4) $$\mathrm{Biodegradability}\;(\%)=\frac{W_{i}-W_{f}}{W_{i}}\times 100\%$$

The electrical properties of the composite films were evaluated using Electrochemical Impedance Spectroscopy (EIS) and Cyclic Voltammetry (CV) techniques (Squidstat Plus, Tempe, AZ, USA) and analyzed via VersaStudio 2.60.6 software. The sample films were securely positioned between two electrode plates. EIS testing was conducted over a frequency range from 10^5^ Hz to 1 Hz with an AC voltage amplitude of 250 mV_rms_ at room temperature, and the Nyquist plot and Bode plot were obtained. The CV test was conducted at a scan rate of 0.1 V/s, running for 3 cycles. Ensuring precise and reliable measurements, the samples were carefully prepared and handled to prevent any surface defects or contamination that could impact the electrical properties. At least three specimens were tested for each composition. The electrical conductivity was calculated using the following equation, where σ represents the electrical conductivity (Ω·cm)^−1^ or (S/cm), L is the sample thickness (cm), |Z| is the calculated impedance magnitude (Ω), A is the electrode cross-sectional area (cm^2^), Z′ is the real part of the complex impedance (Ω), and Z″ is the imaginary part of the complex impedance (Ω).

(5) $$\mathrm{Electrical}\;\mathrm{Conductivity}\;(\sigma )=\frac{L}{|Z|\times A}$$

where |Z| is determined by the following equation:

(6) $$|Z|=\sqrt{{{\left|Z^{\prime }\right|}^{2}+|Z^{\prime \prime }|}^{2}}$$

## 3. Results and Discussion

### 3.1. Fourier Transform Infrared (FTIR) Spectroscopy

**Figure 2:**
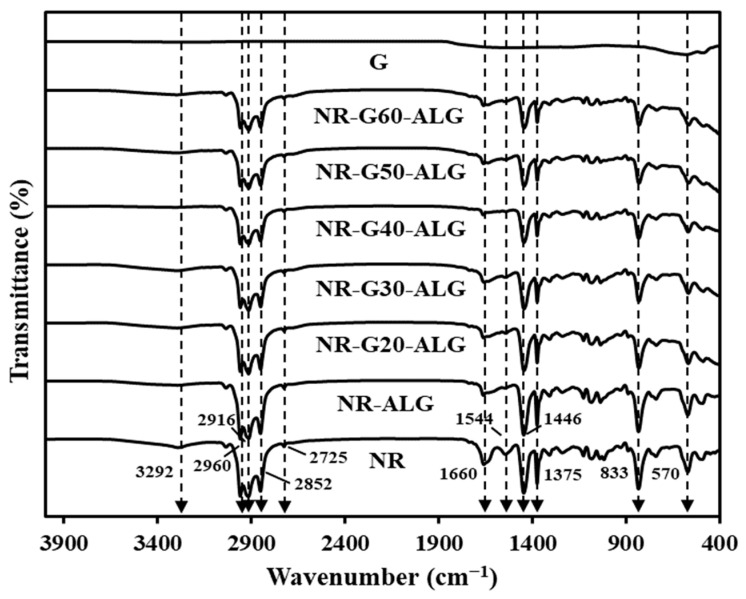
FTIR spectra of NR, G, NR-ALG, and NR-G-ALG composite films.

The FTIR spectrum for NR, G, and NR composite films is illustrated in Figure 2. The neat NR primarily consists of *cis*-1,4-polyisoprene, and its spectrum exhibits distinct peaks at specific wavenumbers. The NR spectrum exhibits characteristic peaks at various wavenumbers, each corresponding to specific chemical functional groups, as follows. The stretching vibrations of the -OH group is at 3292 cm^−1^, the asymmetric stretching vibration of the CH_3_ group is at 2960 cm^−1^, the symmetric stretching vibration of the -CH_2_ group is at 2916 cm^−1^, the symmetric stretching of -CH_2_ is at 2852 cm^−1^, the asymmetrical -CH_3_ overtone deformation is at 2725 cm^−1^, and the C=C stretching is at 1660 cm^−1^ [26,34,35]. The N-H in-plane bending and C-N stretching are attributed to the peptide bonds in proteins, observable at approximately 1541 cm^−1^ [36]. The C-H deformation is at 1446 cm^−1^, the peak at about 1375 cm^−1^ is ascribed to –CH_3_ bending, the C-H out-of-plane bending is at 833 cm^−1^, and the C-C-C in-plane bending is at 570 cm^−1^ [26,32,36]. In contrast, the spectrum of G lacks significant peaks and remains almost entirely flat due to its high-order structure and chemical inertness. The same observation has been previously reported [37,38,39].

Across all the samples of composite films, the primary peaks assigned to the NR chemical structure were present. It was shown that no significant difference was observed in terms of peak numbers among the composites and NR samples. This consistency suggested the absence of active functional groups on the surface of G and indicated a lack of significant chemical interactions among NR, G, and ALG. Notably, the peaks that were indicative of NR behaviors exhibited a decrease in intensity with increasing G loading.

### 3.2. Field Emission-Scanning Electron Microscope (FE-SEM)

**Figure 3:**
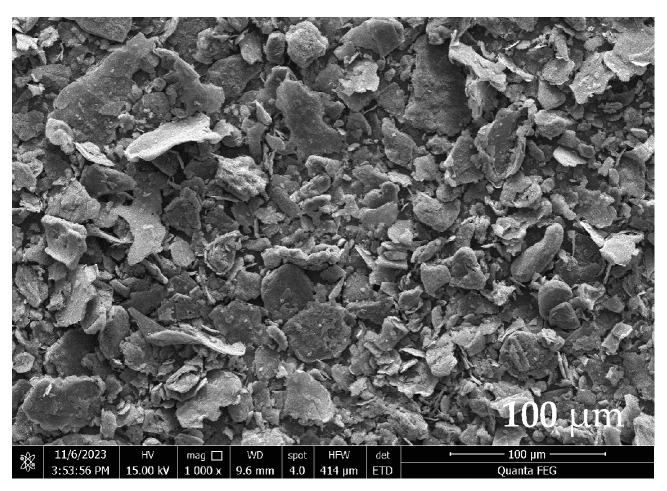
FE-SEM images of graphite (G) platelets.

**Figure 4:**
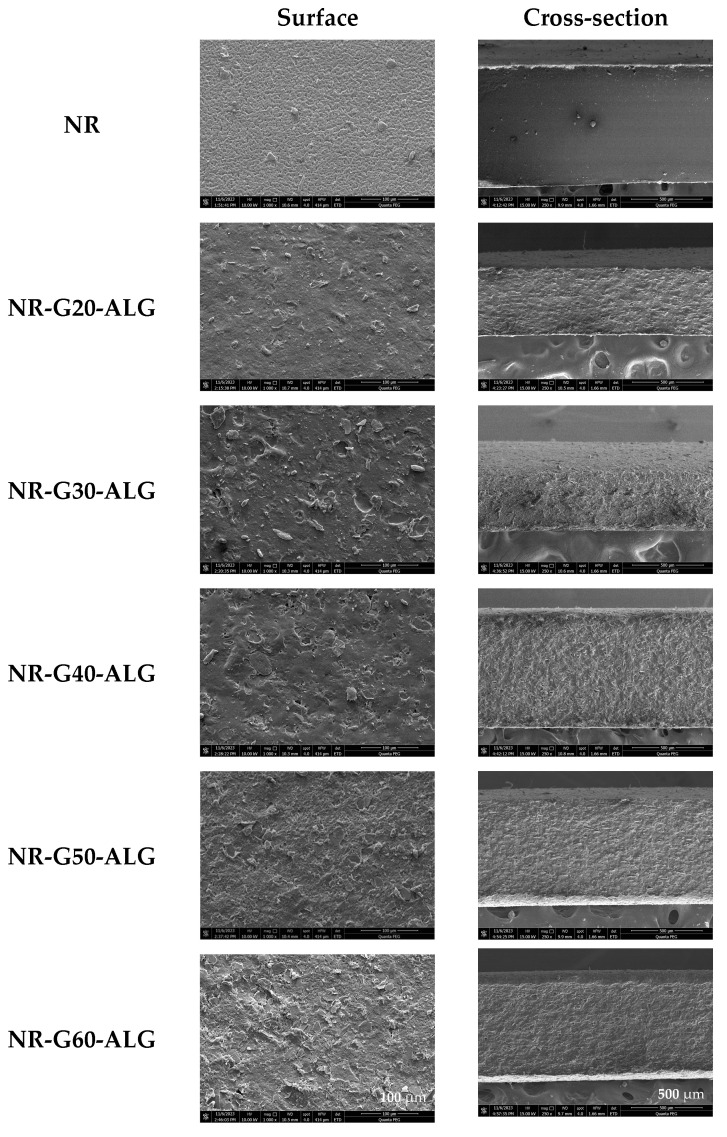
FE-SEM images of the surface morphologies and cross-sections of NR and NR-G-ALG at various loadings of 20 to 60 phr.

FE-SEM analysis was conducted on NR, G, and NR-G-ALG composite films. As shown in Figure 3, G platelets display a platelet-like and planar structure, with an average size of approximately 22.0 ± 7.1 µm (measured by ImageJ program). Figure 4 shows the uniform and smooth texture of the NR films. The NR-G-ALG films exhibited a comparatively rougher surface with a degree of roughness increasing with the higher amount of G loading. Nevertheless, these composite films demonstrated consistent uniformity, with the G platelets evenly dispersed and well-integrated into the NR matrix, as shown in both the surface and cross-sectional views. This indicated the effective dispersion of G platelets facilitated by ALG. The NR-G-ALG films showed the integration of G platelets in the NR matrix without phase separation and agglomeration, suggesting that ALG could serve as a suitable dispersing agent, enhancing the distribution and dispersion of G in the NR matrix. The low-concentration aqueous ALG solution exhibited high viscosity and contained numerous -OH and -COOH groups capable of intercalating into graphene layers [40]. This intercalation may prove beneficial in preventing the agglomeration of G platelets. During the film fabrication process, it was observed that, in the absence of ALG, G platelets faced challenges in achieving uniform dispersion within the NR matrix, and it resulted in the formation of clusters or agglomerates, leading to non-uniform composite films that might impact the overall properties of the composites.

### 3.3. X-ray Diffraction (XRD)

**Figure 5:**
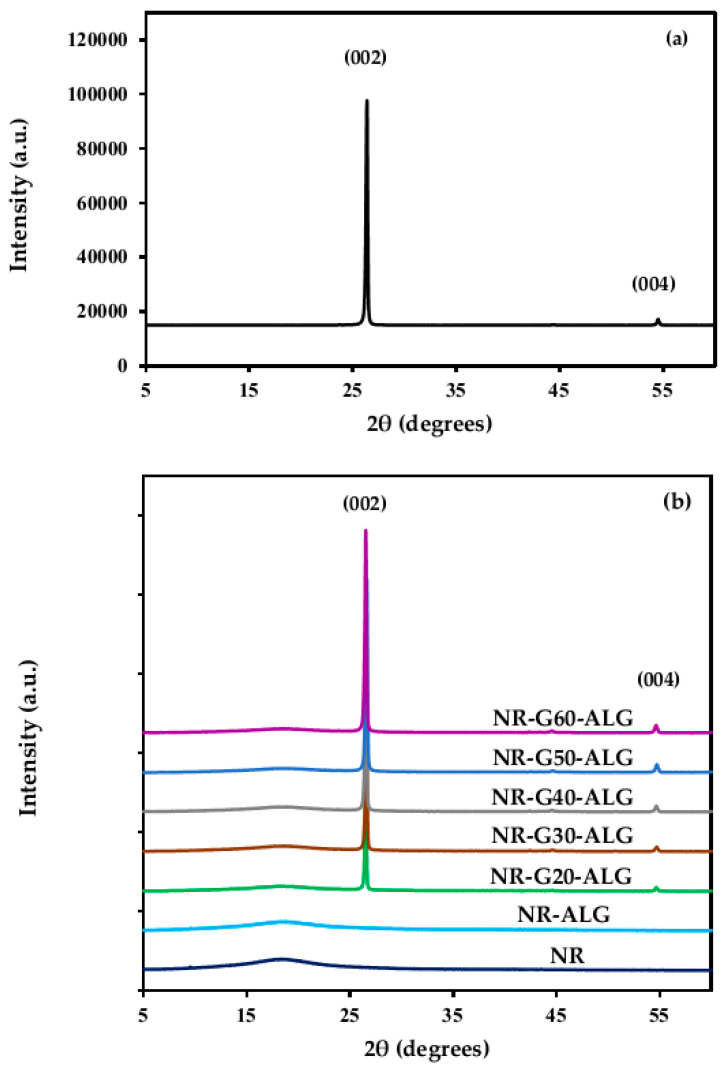
XRD diffractograms of (**a**) G (**b**) NR, NR-ALG, and NR-G-ALG composite films.

In Figure 5, the XRD diffractograms of NR, G, and NR composite films are illustrated. The XRD diffractogram of G platelets exhibited a distinct, intense peak at 2θ = 26.4° and a minor peak at 54.5°, corresponding to the (002) and (004) crystallographic diffraction planes, confirming the highly ordered hexagonal crystalline lattice of G (JCPDS No. 00-041-1487) [41,42]. The neat NR film revealed a broad peak centered around at 2θ = 18°, indicating its amorphous polymer chain structure. The NR-G-ALG composite films displayed diffraction peaks corresponding to both NR and G characteristics. The intensity of the G peak increased with increasing G loading. The degree of crystallinity of the G platelets was determined to be 91.1%, where the degrees of crystallinity of NR composite films with G incorporations at 20, 30, 40, 50, and 60 phr were at 27.8%, 30.7%, 36.6%, 46.8%, and 48.3%, respectively. With the integration of G in the NR matrix, the XRD result showed an increase in the crystallinity of NR composite films related to G concentration.

### 3.4. Mechanical Properties

**Figure 6:**
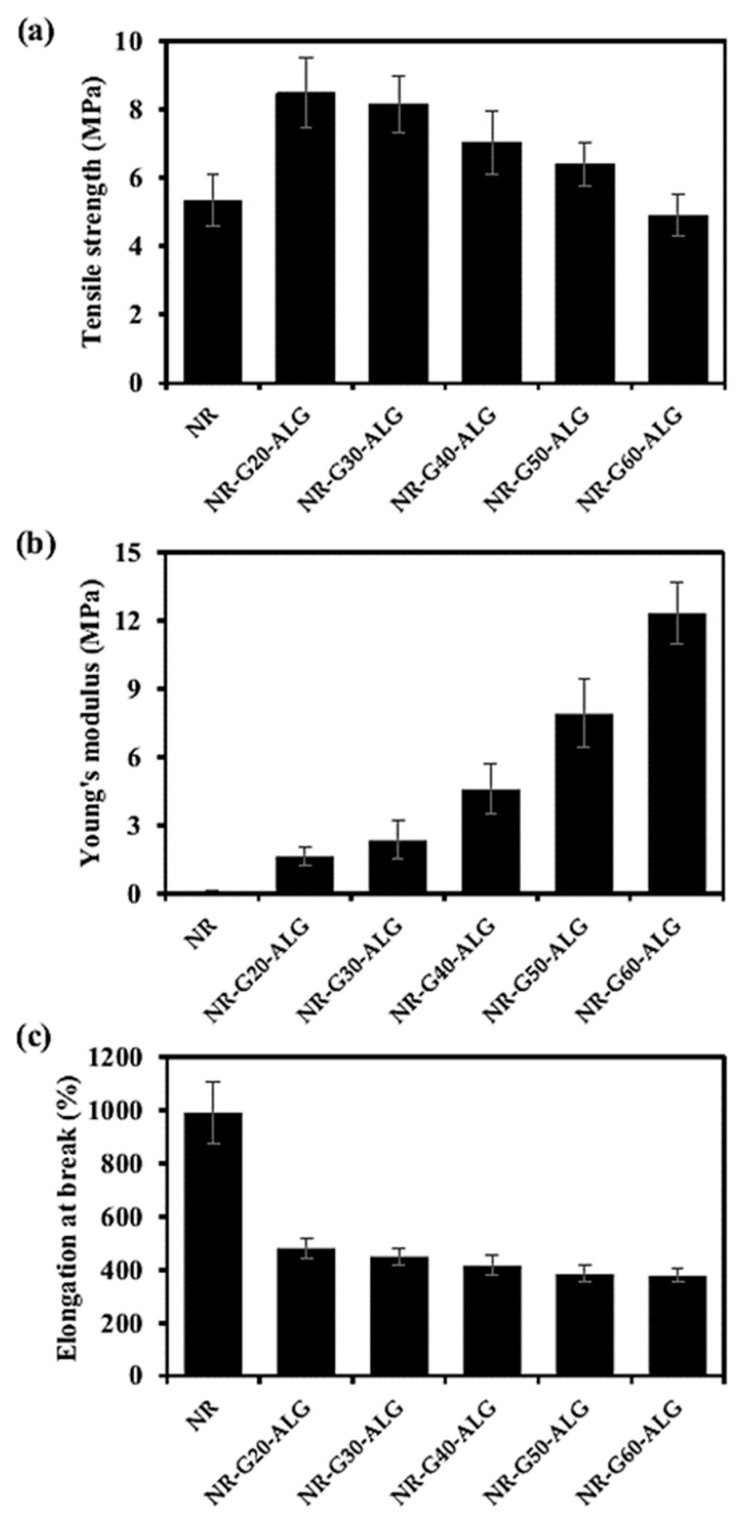
Mechanical properties of NR and NR-G-ALG composite films: (**a**) tensile strength, (**b**) Young’s modulus, and (**c**) elongation at break.

The mechanical properties of the NR composite films, reinforced with various loadings of G and the neat NR film, were analyzed in Figure 6. The neat NR film exhibited tensile strength, Young’s modulus, and elongation at break values of 5.33 MPa, 0.12 MPa, and 992.4%, respectively. The values of tensile strength and Young’s modulus were significantly enhanced with the introduction of G platelets. For instance, the tensile strength of the NR film reinforced with 20 phr of G increased to 8.47 MPa, representing a substantial improvement of 1.6-fold over the neat NR film. However, with the increased incorporation of G beyond 20 phr, the tensile strength gradually decreased. This could be attributed to a reduction in the thickness of the NR matrix interlayers, which at higher G loadings might become insufficient to adequately coat all available filler surfaces [43]. Furthermore, because G possesses a high density and surface area [18], it is prone to agglomeration, especially at very high loading, which could result in reduced interfacial adhesion between the filler and matrix. Additionally, the structure of reinforcing fillers can have an effect on brittleness in fracture and reduced fracture toughness, thus may have a potential impact on the material’s mechanical properties [44]. Under a study on polysulfone composites reinforced with different types of graphite, it was revealed that the initial addition of artificial graphite (AG) up to a certain amount could improve the tensile strength of the composite. However, when the AG loading exceeded 50% by weight, the tensile strength of the composite decreased, and further additions of AG continued to reduce the tensile strength [43].

Young’s modulus quantifies a material’s stiffness and its resistance to deformation under stress. Graphite, as a carbonaceous micro filler, has been known for its rigidity and stiffness [45]; therefore, the addition of G resulted in a sequential increase in Young’s modulus of the composite films. The maximum Young’s modulus value was achieved with NR composite films with G incorporation at 60 phr, reaching 12.33 MPa, or approximately 100 times greater than that of the neat NR film, which exhibited a low value of Young’s modulus at only 0.12 MPa.

Due to the inherent elasticity and flexibility of NR, its elongation at break is exceptionally high. Therefore, the addition of a stiff and rigid filler resulted in a reduction in the elongation at the break of the NR composite. The decrease in elongation at break can be attributed to the heightened rigidity introduced by the presence of G, which restricts the material’s capacity to stretch and deform before reaching the point of failure. It was shown that the elongation at break of the NR composite films with G incorporations varied between 480% and 377%, depending on the loading content of G, whereas the elongation of the neat NR film was 992%.

Our observation is aligned with prior investigations. The reinforcement of thermally reduced graphite oxide (RGOT) in the NR matrix showed increased tensile strength upon incorporating RGOT up to a certain amount [45]. However, the higher RGOT loading beyond a specific loading threshold (10 phr) led to a gradual decline in tensile strength and a reduction in the composite’s elasticity [39]. Under the reinforcement of NR with carboxylated MWCNTs dispersed with sodium dodecyl sulfate (SDS), a 10-fold increase in Young’s modulus and a 2-fold rise in tensile strength was obtained when compared to the neat NR [46]. The enhancement in mechanical strength could be related to the creation of a rigid network of reinforcing fillers within the NR latex structure, which might be facilitated by cross-linking via the functional groups found at the surface of the CNTs and the organic molecules present in the NR latex [46].

### 3.5. Thermal Properties

**Figure 7:**
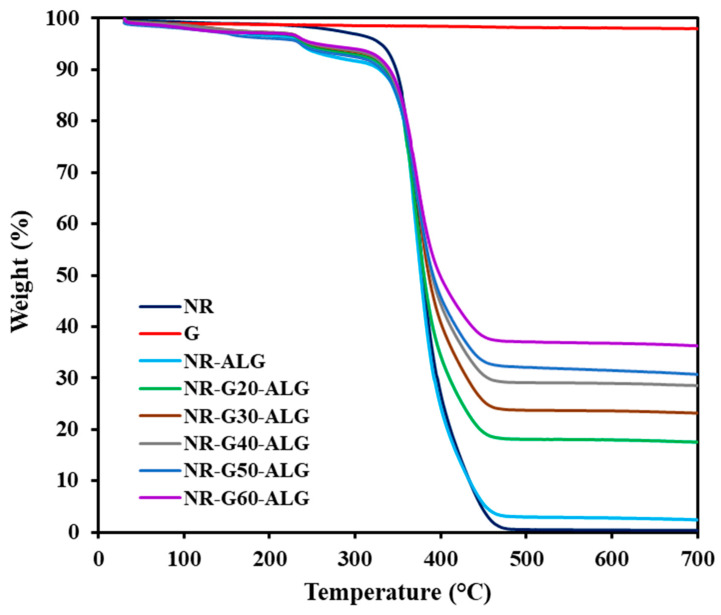
TGA curves of the NR, G, NR-ALG, and NR-G-ALG composite films.

**Figure 8:**
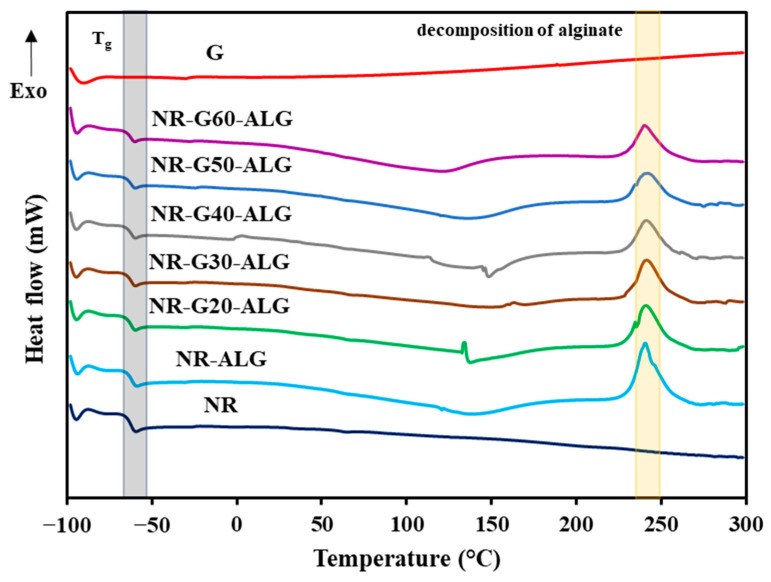
DSC curves of the NR, G, NR-ALG, and NR-G-ALG composite films.

Thermal stability is a crucial characteristic of electronic applications and materials, influencing both their processing and potential utility in high-temperature applications. The thermal properties of NR, G, and NR-G-ALG composite films were explored using TGA and DSC, as depicted in Figure 7 and Figure 8, respectively. Table 1 presents thermal data for all samples, including decomposition temperatures at 5%, 10%, and 50%wt (T_5%_, T_10%_, and T_50%_) and residual mass at 700 °C.

The addition of ALG influences the decomposition temperature values, as its lower degradation temperature precedes that of NR. In the composite films, decomposition temperatures demonstrate an upward trend with increasing G content. Additionally, the incorporation of G contributes to an increased residual mass at 700 °C, highlighting its role in enhancing the thermal stability of the composite films. The crystalline nature of G platelets introduces a barrier effect, enhancing resistance to thermal degradation and impeding the release of decomposition products into the gas phase from the polymer [47].

Figure 7, which presents TGA, reveals that NR experienced an initial weight loss within the temperature range of 330–480 °C. The total weight loss of NR reached approximately 99.63%, indicating nearly complete degradation. This substantial weight loss can be attributed to NR’s poor thermal resistance. In contrast, The TGA curve for G confirmed its exceptional thermal stability, with the weight remaining at approximately 97.98%, even when exposed to temperatures as high as 700 °C.

For NR-ALG and NR-G-ALG composite films, the thermal degradation of the composite exhibited a 2-step process. The first degradation occurred at T_d_ around 220 °C, likely due to the presence of ALG, and underwent a decomposition process involving the removal of moisture and surface-bound water [48]. The second weight loss occurred within the range of 330–480 °C, corresponding to NR degradation, as previously mentioned. The total remaining weight of NR-G-ALG ranged from 17.54 to 36.27%, related to G content in the composite films. Therefore, the incorporation of G led to higher residue levels, owing to its exceptional thermal resistance of G even at temperatures exceeding 700 °C.

The DSC thermograms presented in Figure 8 depict the thermal characteristics of NR, G, NR-ALG, and NR-G-ALG composite films. The thermogram corresponding to the NR film highlights its amorphous nature, with a glass transition temperature (T_g_) of −63.5 °C. This transition temperature marks the point at which the material transforms from a rigid to a more flexible state, reflecting the NR’s inherent properties. In the case of G, there are no distinct thermal peaks observed in its thermogram. The absence of significant peaks in the thermogram points to the exceptional thermal stability exhibited by G. Notably, upon analyzing the thermograms of all NR-G-ALG composite films, no significant change in T_g_ was observed. The broad endothermic peaks observed in the temperature range of 80–150 °C were assigned to the evaporation of water molecules associated with the ALG polymer [49]. Additionally, the exothermic peaks, centered at around 240 °C, were attributed to the decomposition of the alginate polymers, resulting in the formation of carbonized residue [50].

### 3.6. Water Absorption Capacity (WAC)

**Figure 9:**
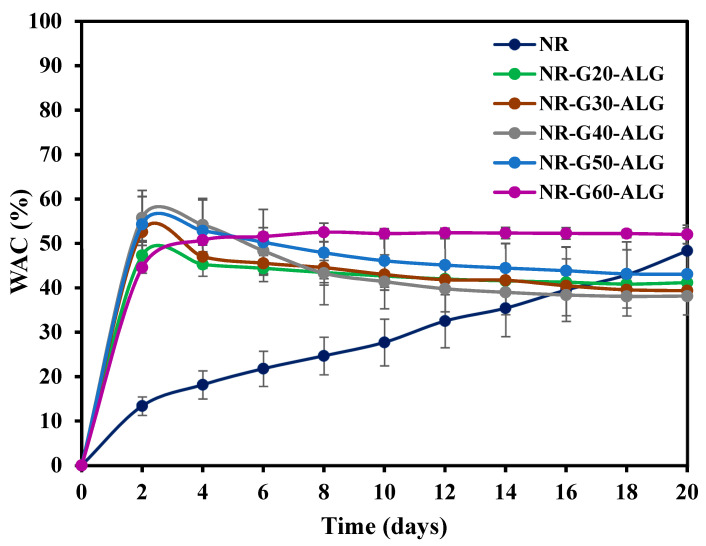
The water absorption capacity results of the NR and NR-G-ALG composite films.

The water absorption capacity (WAC) of NR and NR composite films is presented in Figure 9. In the case of the neat NR film, a gradual increase in WAC is observed over a 20-day period. However, with the addition of ALG, a polar molecule known for its hydrophilic and water-retention properties, a remarkable change in the water affinity of the composite films is evident. Therefore, the initial water uptake rate of NR composite films in the first 2 days is much higher than that of neat NR. After 2 days of water immersion, the NR-G-ALG films exhibited nearly constant levels of water absorption.

The water absorption behavior in filled polymer composites depends on various factors, including temperature, filler properties (such as functionality, polarity, and specific surface area), filler loading and morphology, filler volume fraction, and porosity [51,52]. After 20 days of immersion, the WAC for NR composites reinforced with varying G loadings ranged from 38% to 52%. The NR composite films with G incorporations at 60 phr demonstrated the highest WAC. Many studies have investigated the water absorption of graphite or graphene-filled polymer composites and reported that the WAC tended to decrease with increasing graphite or graphene content. This effect might be attributed to the barrier created by the platelet structure of the fillers, which hinders water transportation, as well as the hydrophobic nature of the fillers [53,54,55].

In this study, after the equilibrium, the WAC of NR-G-ALG composite films loading with G platelets from 20 to 50 phr demonstrated slightly lower as compared to that of NR film (WAC ≈ 48%). However, with the incorporation of G at 60 phr, the WAC of the film increased to approximately 52%, which was slightly higher than that of NR film. Some previous studies also reported a similar result. For instance, Alo and Otunniyi studied graphite-filled high-density polyethylene (HDPE) and epoxy composites and found that the WAC increased with high graphite content, which could be attributed to poor interfacial adhesion [56]. Additionally, it was reported that an increase in WAC in graphite-filled polymers could be due to increased porosity and holes present [57,58]; this aligns with the voids observed from the FE-SEM image of composite films at high G loading of NR-G60-ALG with an approximate size of holes of 1.65 ± 0.66 µm (figure not shown). The high loading of G in the NR matrix appears to augment the presence of voids in the composite, potentially explaining the increase in WAC.

### 3.7. Toluene Uptake

**Figure 10:**
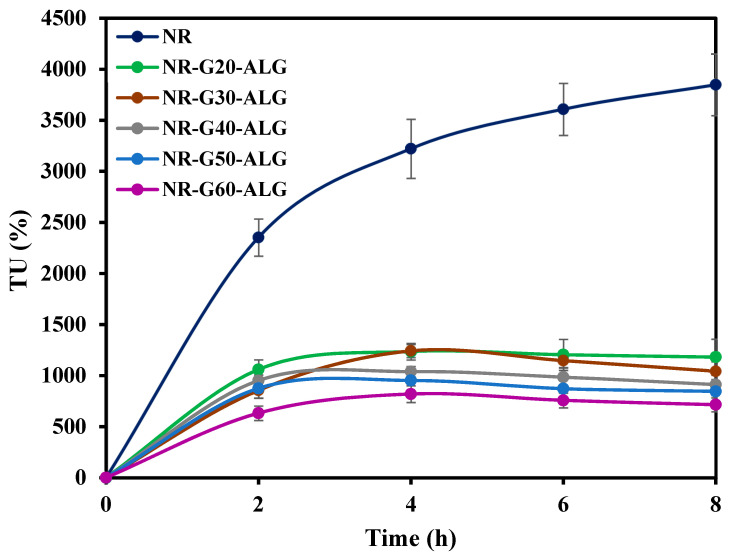
The toluene uptake results of the NR and NR-G-ALG composite films.

The toluene uptakes of NR and NR composite films following an 8 h immersion in toluene are presented in Figure 10. The NR film demonstrated the highest toluene uptake, reaching 3848%, and this uptake increased further with a longer immersion time. Toluene and NR are both non-polar substances; therefore, NR films exhibit a strong affinity for this solvent. The high uptake rate and poor chemical resistance of NR have been previously reported by many studies.

In the case of NR-G-ALG films, the toluene uptake was significantly lower than that of the NR film. Although some slight swelling occurred, the composite films were able to maintain their structural integrity. Moreover, with longer immersion in toluene, NR-G-ALG films exhibited a consistently lower toluene uptake. As the G loading increased, the toluene uptake decreased remarkably. The composite film with the lowest toluene uptake was NR-G60-ALG, which exhibited an uptake of 714.9% after the 8 h toluene immersion. NR-G60-ALG showed a much lower toluene uptake at approximately 18.6% of that of NR film. The decrease in toluene uptake could be attributed to the chemical stability of graphite. Additionally, the presence of the filler network reduced the mobility of the rubber chains, resulting in a slower swelling process within the rubber network [35]. This reduction was further facilitated by the formation of a framework-like network structure involving the fillers, effectively limiting the permeation of toluene into the rubber chains [59]. This phenomenon aligns with the findings of a similar study by Ismail and Khalaf, who explored graphite-filled styrene–butadiene rubber (SBR) composites. Their research revealed that graphite-filled SBR exhibited superior toluene resistance, and as the concentration of graphite increased, swelling decreased due to improved graphite dispersion and strong interactions between graphite and the polymer matrix [60]. Additionally, ALG is a polar molecule. The incorporation of polar components could indeed play a crucial role in reducing the toluene uptake rate and absorption capacity [26]. This observation also implies that the composite films achieve good dispersion and distribution of G within the NR matrix.

### 3.8. Biodegradation in Soil

**Figure 11:**
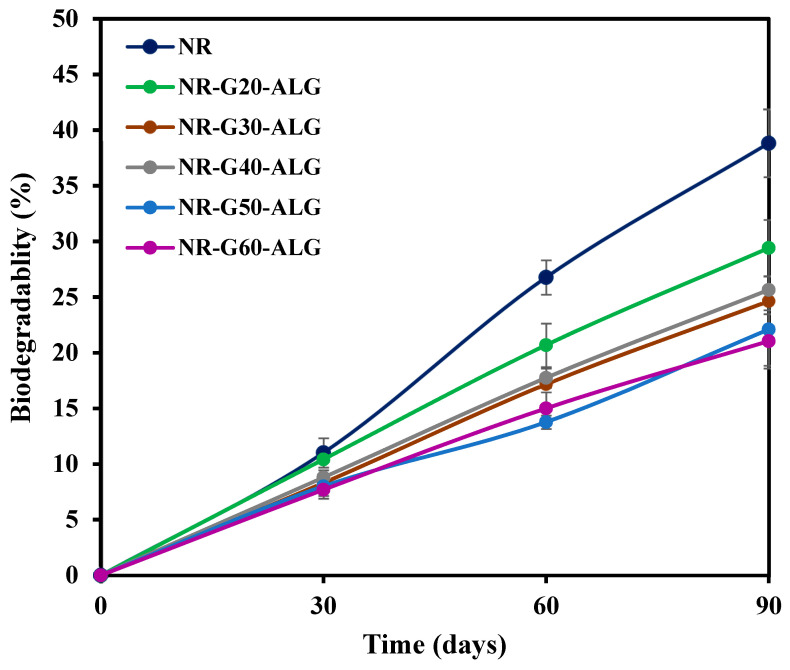
Biodegradation test results of the NR and NR-G-ALG composite films.

Biodegradability is a crucial property of environmentally friendly materials. Biodegradation in soil is driven by microorganisms that break down complex organic compounds into simpler molecules. Ultimately, this process results in the production of water, carbon dioxide (under aerobic conditions), or methane (under anaerobic conditions) [61,62]. Figure 11 illustrates the relationship between biodegradability and the duration of burial. The NR and NR composite films underwent a 90-day burial period. Because of its intrinsic amorphous nature, NR readily accommodates microorganisms within its structure. NR film is a bio-based and biodegradable product, which could be broken down by numerous bacteria and fungi [63]. Following over 90 days of burial, the neat NR film exhibited a progressive increase in biodegradation, reaching a value of 38.8%.

With the incorporation of G platelets, which are a highly stable carbon allotrope known for its crystallinity and non-toxic nature in the environment, the weight loss of NR-G-ALG composite films was significantly lower compared to that of the neat NR film. However, the weight loss rate also increased over time. After 90 days of burial, NR-G60-ALG demonstrated the lowest weight loss at 21.0%, whereas NR-G20-ALG exhibited higher weight loss at 29.4%. The incorporation of G into the composite films is believed to contribute to an increase in their degree of crystallinity. In general, crystalline polymer tends to have denser molecular packing, thus slowing down its rate of breakdown [64].

**Figure 12:**
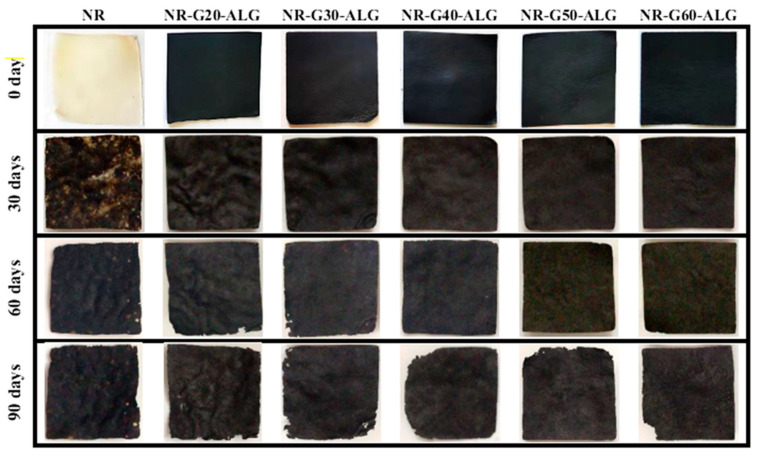
The visual analysis of biodegradation test of NR and NR-G-ALG composite films for the duration of 90 days.

A visual analysis of NR and NR composite films is presented in Figure 12. It was observed that the NR films displayed black spots after being buried in the soil, indicating the likely influence of microbial activity and environmental factors during the burial. Over time, the composite film developed wrinkles, and certain sections of the film degraded. Some disintegration into small pieces was also observed, indicating microbial activity’s role in degrading the film. This result underlines the biodegradation of NR composite films, which is favorable for environmentally friendly and sustainable applications.

### 3.9. Electrical Properties

**Figure 13:**
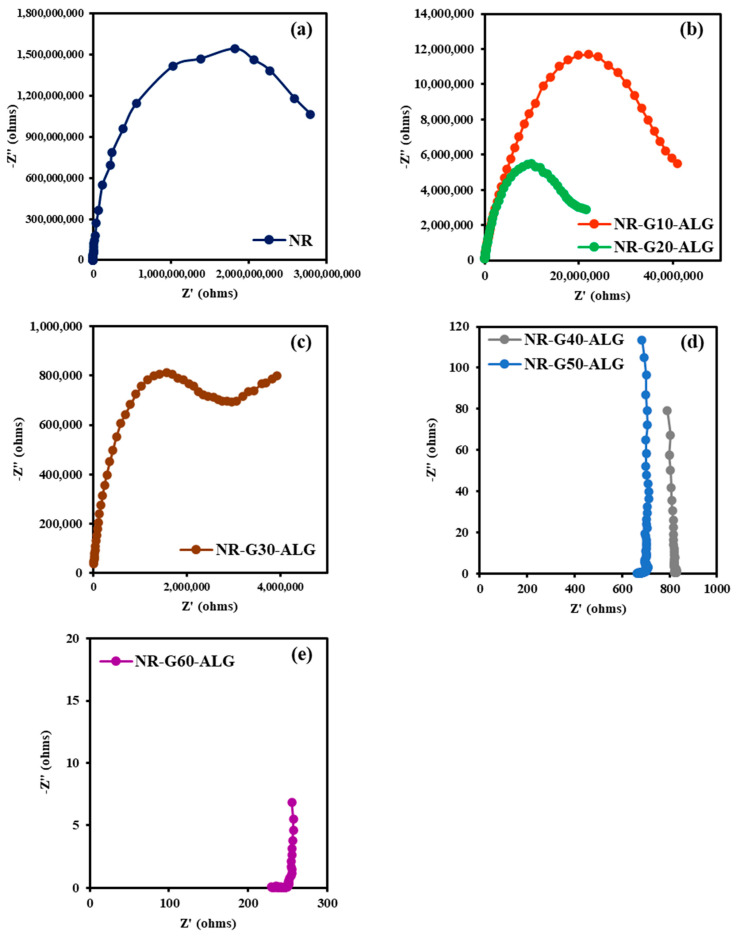
Nyquist plots of NR and NR-G-ALG across a frequency range of 10^5^ Hz to 1 Hz, exhibited within various impedance ranges: (**a**) NR, (**b**) NR-G10-ALG and NR-G-20-ALG, (**c**) NR-G30-ALG, and (**d**) NR-G40-ALG, NR-G50-ALG, and (**e**) NR-G60-ALG.

Electrochemical impedance spectroscopy (EIS) assessed the electrical conductivity of NR and NR composite films across a frequency range from 10^5^ Hz to 1 Hz. NR-G10-ALG films were intentionally fabricated by using G filler for improved electrically conductive properties. The Nyquist plots in Figure 13 depict the relationship between real and imaginary impedances of fabricated NR and NR composite films, exhibiting behavior dependent on G loading content. Smaller semicircle diameters indicate higher electrical conductivity due to lower impedance magnitude [65].

The incorporation of G platelets into NR composite films is expected to enhance their electrical conductivity owing to G’s intrinsic electrical properties. This improvement is attributed to the even distribution of π orbitals and free electrons across the G layers, along with the influence of high crystallinity, which significantly contributes to the enhanced electrical and thermal conductivity of the composite films.

The Nyquist plots exhibited distinct behaviors corresponding to various G loading levels. In the NR plot, the largest semicircle, signifying its inherent electrical insulation properties, was observed. The calculated electrical conductivity was 1.43 × 10^−11^ S/cm, which was the lowest among all samples. With the increase in G loading, the semicircle in the composite films becomes smaller due to the introduction of a conductive filler. The calculated electrical conductivities were 1.14 × 10^−9^, 5.59 × 10^−9^, and 8.55 × 10^−9^ S/cm for NR composites with G loading of 10, 20, and 30 phr, respectively.

As the G loading continues to increase, reaching 40 and up to 60 phr, the signal shifts from a “semicircle” to a “linear-like” graph characteristic, where the intercept of the plot on the real axis representing the impedance, denoting the bulk resistance [66]. This transition suggests a significant movement toward conductivity within the composite, as revealed by the observed electrical behavior. The incorporation of G notably lowers the x-intercept of the plot, signifying an improvement in conductivity. The measured electrical values for NR composites with G loadings of 40, 50, and 60 phr were 2.77 × 10^−5^, 5.26 × 10^−5^, and 2.18 × 10^−4^ S/cm, respectively. Remarkably, a significant increase in conductivity is observed between G loadings of 30 and 40 phr. This observation suggests that the percolation threshold of G within the NR matrix falls approximately within this range. It indicates a conductivity increase in approximately four orders of magnitude compared to the 30 phr loading and roughly six orders of magnitude compared to the neat NR. The enhanced conductivity falls within the range observed for semiconducting materials [67].

**Figure 14:**
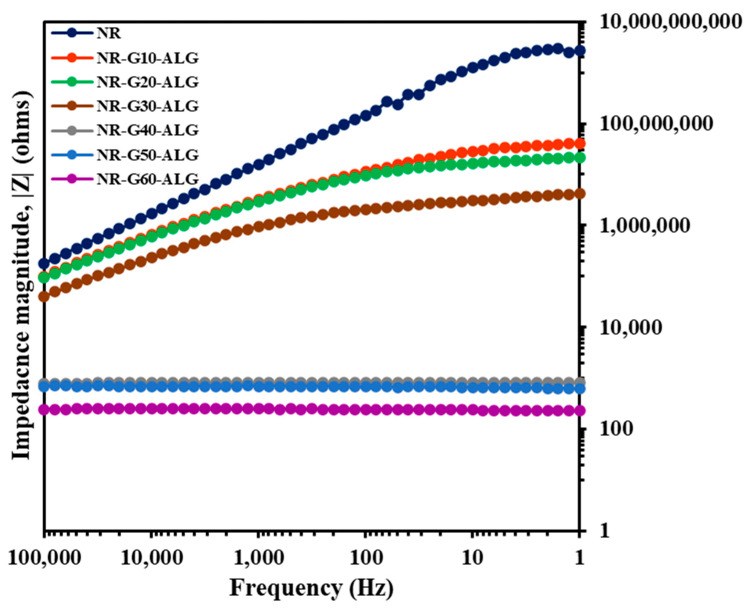
The Bode plot depicts the impedance magnitude of NR and NR-G-ALG with G loading ranging from 10 to 60 phr over a frequency range of 10^5^ Hz to 1 Hz.

In Figure 14, the Bode plot exhibits a linear relationship between impedance magnitude and frequency. With an increase in the incorporation of G loading, there is a consecutive decrease in impedance magnitude. Among the samples, NR-G60-ALG demonstrated the lowest impedance magnitude across all frequency ranges. In samples with G loading higher than 30 phr, the linear behavior of impedance no longer follows the previous crescent trend observed in those with lower G loadings and the neat NR. In fact, it tends toward a plateau, appearing as a horizontal line with no slope. This characteristic indicates that the impedance is no longer influenced by frequency in lower frequencies, suggesting the achievement of electrical percolation [65].

**Figure 15:**
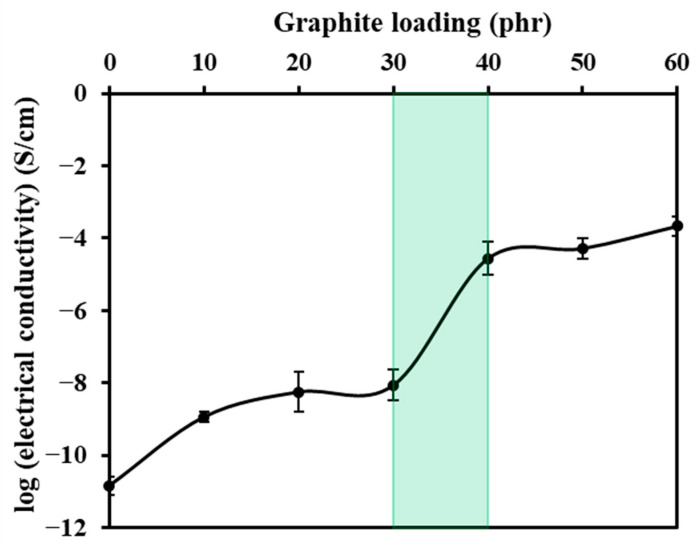
The relationship between log (electrical conductivity) and G loading, highlighting the percolation threshold within the graphite loading range of 30 to 40 phr.

Figure 15 displays the graph illustrating the relationship between electrical conductivity and G loading, showcasing a distinctive S-shape percolation transition [45]. The conductivity rises proportionally with increasing G loading until a certain threshold, beyond which only moderate increases are evident with additional G incorporation. This behavior indicates the development of a continuous conductive network after surpassing the percolation threshold, facilitating efficient electron transport throughout the composite. These conductive networks form as a result of the integrated conductive fillers. At the critical point of filler concentration, the material transitions from an insulating to a conducting state. The initial development of conduction pathways leads to a drastic increase in electrical conductivity.

**Figure 16:**
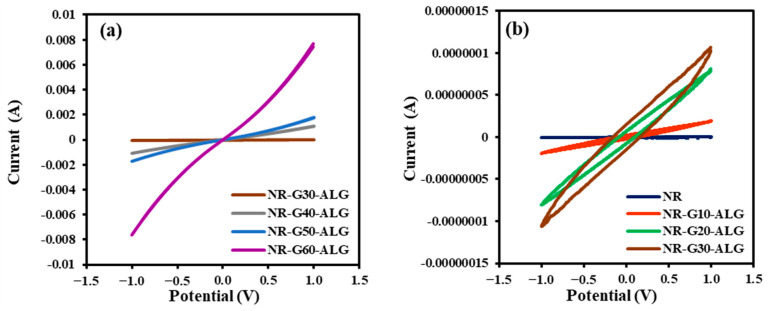
The cyclic voltammetry (CV) measurement of NR and NR-G-ALG, taken at a scan rate of 0.1 V/s, displays the graph representing the 2^nd^ cycle (**a**) at G loading above percolation threshold and (**b**) at G loading below percolation threshold.

For CV measurements (Figure 16), the NR and NR composite films were assessed within a potential range of −1.0 V to 1.0 V. In NR and NR-G-ALG composites with low G loading (up to 30 phr), the closed-loop rectangular shape in the CV graphs suggests non-linear electrical behavior, resembling capacitance behavior. This behavior may be linked to incomplete conductive pathways, resulting in a non-uniform electrical response. However, as the G loading increases, there is a noticeable increase in the slope of the graphs. Beyond the percolation threshold, the observed linear-like behavior in the CV graphs may indicate the establishment of a continuous conductive network within the composite. This network could facilitate efficient electron transport, leading to more linear and predictable electrical responses.

## 4. Conclusions

Unvulcanized NR composites reinforced with synthetic graphite platelets (G) at different loadings from 20 to 60 phr were successfully fabricated using sodium alginate as a dispersing and thickening agent. FTIR analysis indicated no chemical interaction between NR, G, and ALG. FE-SEM images depict the effective dispersion of G without any phase separation in the NR matrix, evident from both surface and cross-sectional perspectives. The reinforcement with G resulted in an increased degree of crystallinity compared to the inherent amorphous nature of NR. NR-G-ALG films displayed remarkably improved tensile strength and Young’s modulus, whereas elongation at break decreased. The maximum tensile strength of 8.5 MPa was attained when incorporated with G at 20 phr, while the highest Young’s modulus of 12.3 MPa was obtained when incorporated with G at 60 phr. The NR composite films reinforced with G exhibited significantly enhanced chemical resistance, as evidenced by a toluene uptake that was only 18.6% of that observed in the neat NR film. The biodegradation test in soil over a three-month period demonstrated that the composite films achieved a biodegradation value ranging from 21.0% to 29.4%. Additionally, the electrical conductivity of the NR composite films exhibited a remarkable enhancement, reaching an approximate seven-order of magnitude increase compared to that of the neat NR film. The maximum conductivity at 2.18 × 10^−4^ S/cm was obtained from the NR composite film with G loading at 60 phr. The percolation threshold was identified to be between 30 and 40 phr of G loading. This research underscores the noteworthy enhancements in the degree of crystallinity, mechanical properties, chemical resistance, and electrical conductivity of NR composites reinforced with G platelets, indicating their promising potential as a conductive substrate for electronic applications.

## Figures and Tables

**Table 1:** Thermal data for NR, G, and NR-G-ALG composite films.

Sample	Graphite Loading (phr)	T_5%_ (°C)	T_10%_ (°C)	T_50%_ (°C)	Residual Mass at 700 °C (wt.%)
NR	-	330.1	347.8	379.3	0.37
NR-ALG	-	236.7	328.8	376.7	2.41
NR-G20-ALG	20	242.5	335.6	380.3	17.54
NR-G30-ALG	30	248.2	338.3	385.9	23.18
NR-G40-ALG	40	250.2	337.7	389.7	28.54
NR-G50-ALG	50	246.6	331.2	391	30.70
NR-G60-ALG	60	257.2	340.4	399.1	36.27
G	100	-	-	-	97.98

## Data Availability

The supporting data for the results of this study can be obtained upon request from the corresponding author.
